# *Walterinnesia aegyptia *venom combined with silica nanoparticles enhances the functioning of normal lymphocytes through PI3K/AKT, NFκB and ERK signaling

**DOI:** 10.1186/1476-511X-11-27

**Published:** 2012-02-15

**Authors:** Gamal Badr, Mohamed K Al-Sadoon, Ahmed M El-Toni, Maha Daghestani

**Affiliations:** 1 Deanship of Scientific Research, King Saud University, P.O. Box 2454, Riyadh 11451, Saudi Arabia; 2 Zoology Department, College of Science, King Saud University, Riyadh, Saudi Arabia; 3 King Abdullah Institute for Nanotechnology, King Saud University, Riyadh, Saudi Arabia; 4 Zoology Department, Faculty of Science, Assiut University, 71516 Assiut, Egypt

**Keywords:** Cytoskeleton, Growth arrest, Lymphocytes, Nanoparticles, Proliferation, Cell signaling, Snake venom

## Abstract

**Background:**

The toxicity of snake venom varies over time in some species. The venom of newborn and small juvenile snakes appears to be more potent than adults of the same species, and a bite from a snake that has not fed recently, such as one that has just emerged from hibernation, is more dangerous than one that has recently fed due to the larger volume of venom injected. Therefore, the potency of a snake's venom is typically determined using the LD_50 _or IC_50 _tests. In the present study, we evaluated the anti-tumor potential of snake venom from *Walterinnesia aegyptia *(WEV) on the human breast carcinoma cell line MDA-MB-231, as well as its effect on the normal mice peripheral blood mononuclear cells (PBMCs).

**Results:**

This venom was used alone (WEV) or in combination with silica nanoparticles (WEV+NP). The IC_50 _values of WEV alone and WEV+NP in the MDA-MB-231 cells were determined to be 50 ng/ml and 20 ng/ml, respectively. Interestingly, at these concentrations, the venom did not affect the viability of normal human PBMCs. To investigate the *in vivo *effects of this venom further, three groups of mice were used (15 mice in each group): Group I was the control, Group II was subcutaneously injected with WEV, and Group III was injected with WEV+NP. Using flow cytometry and western blot analysis, we found that the blood lymphocytes of WEV-injected mice exhibited a significant increase in actin polymerization and cytoskeletal rearrangement in response to CXCL12 through the activation of AKT, NF-κB and ERK. These lymphocytes also showed a significant increase in their proliferative capacity in response to mitogen stimulation compared with those isolated from the control mice (P < 0.05). More importantly, in the WEV+NP-treated mice, the biological functions of normal lymphocytes were significantly (P < 0.05) enhanced in comparison with those of WEV-treated mice.

**Conclusion:**

Our data reveal the unique biological effects of WEV, and we demonstrated that its combination with nanoparticles strongly enhanced these biological effects.

## Background

Cell proliferation is a basic biological process that occurs continuously in higher organisms in response to changes in their external and internal environments. In the immune system, lymphocyte proliferation is an important parameter indicating the status of the body's defenses [1]. Recent evidence from both animal and human studies further supports the concept that lymphopenia can drive homeostatic proliferation and the development of autoimmune disease [2]. The uncontrolled proliferation of cells leads to cancer metastasis; therefore, several studies have focused on inhibiting the proliferative capacity of cancer cells using various drugs. Directional cell migration is an integral component of cancer cell invasion during metastasis and involves changes in the cytoskeleton and cell adhesion [3]. The migration of cells through an extracellular matrix is a multistep process that begins with the extension of lamellipodia, cell-surface protrusions comprised of actin filaments, which are anchored to the underlying substratum by small, integrin-dependent focal adhesions. In both normal and cancer cells, the polymerization of actin pushes against the plasma membrane and provides the force for forward movement. Within the cell body, actin stress fibers generate contractile forces by pulling against focal adhesions to induce retraction at the rear of the cell membrane. The bundling of actin filaments into stress fibers clusters and activates integrins, leading to the formation of new focal adhesions [4]. In normal cells, several transcription factors are activated following cell stimulation, leading to cytoskeletal rearrangement and proliferation. The extracellular-signal-regulated kinase (ERK), NF-κB and AKT signaling pathways are major determinants in the control of diverse cellular processes in normal lymphocytes such as proliferation, survival, differentiation and motility. Mechanisms for the inhibition of these pathways thus present targets for cancer therapy [5]. Additionally, previous studies have reported that the constitutive activation of NF-κB in human melanoma cells is linked to the activation of AKT kinase, suggesting that the activation of AKT may be an early marker of tumor progression in melanoma [6] and that inhibitors of NF-κB activation can block the neoplastic transformation response [7,8].

Natural products are well recognized as sources for drugs in several human ailments, including cancers. Despite the discovery of many drugs of natural origin, the search for new anticancer agents remains necessary to increase the number of available options and to identify less toxic and more effective drugs [9]. Snake venom is a complex mixture of many substances with a wide spectrum of biological activities including toxins, enzymes, growth factors, activators and inhibitors. Natural toxins, especially sub lethal doses of snake venom, have shown the potential to reduce the size of solid tumors and block angiogenesis [10]. Nanoparticles carrying chemical therapeutics have shown great promise in treating cancer patients. When loaded with anticancer agents, nanoparticles can successfully increase drug concentrations in cancer tissues and act at the cellular level, enhancing antitumor efficacy. The nanoparticles can be endocytosed and/or phagocytosed by cells, with resulting cell internalization of the encapsulated drug [11].

Few studies have investigated the effects of snake venom in combination with nanoparticles on normal and cancer cells. Therefore, in the present study, we investigated the effects of *Walterinnesia aegyptia *venom (WEV), alone and in combination with silica nanoparticles (WEV+NP), on the survival of a human breast carcinoma cell line (MDA-MB-231) and human peripheral blood mononuclear cells (PBMCs) *in vitro *and the *in vivo *effects of WEV and WEV+NP on mouse lymphocytes.

## Materials and methods

### Preparation of Walterinnesia aegyptia venom

*Walterinnesia aegyptia *snakes were collected from the central region of Saudi Arabia. The snakes were kept in a serpentarium in the Zoology Department, College of Science, King Saud University. The snakes were warmed daily using a 100-watt lamp for nine hours, and water was always available. The snakes were fed purpose-bred mice every 10 to 14 days. The venom was milked from adult snakes, lyophilized and reconstituted in 1X phosphate-buffered saline (PBS) prior to use.

### Combination of snake venom with silica nanoparticles

A total of 25 mg of mesoporous silica nanoparticles was added to a solution of 50 mg/ml venom in water. The suspension was stirred for 2 hour; the evaporation of water was prevented. The mesoporous silica nanoparticles loaded with venom were recovered using high-speed centrifugation and dried in a vacuum oven at 60°C.

### Cell culture and reagents

Human MDA-MB-231 breast cancer cells were obtained from Dr. Douaa Sayed at Assiut University, Egypt and maintained in a culture medium consisting of MEM supplemented with 10% heat-inactivated fetal bovine serum (FBS, EuroClone, Life Science Division, Milan, Italy). The anti-proliferative effect of WEV and WEV+NP on MDA-MB-231 cells was determined using the 3-(4, 5-dimethylthiazol-2-yl)-2, 5-diphenyltetrazolium bromide (MTT) uptake method. The cells were plated at 1 × 10^6 ^cells/ml in 2 ml of culture medium in six-well Costar plates (Corning, Corning, NY). The cells were treated with different concentrations of WEV or WEV+NP for 1, 2, 6, 12, 24 or 48 h, and cytotoxicity was expressed as a relative percentage of the OD values measured in the control and WEV- and WEV+NP-treated cells. The experiments were repeated using human peripheral blood mononuclear cells (PBMCs). Morphological changes were observed after exposure to WEV and WEV+NP using a phase-contrast inverse microscope (Olympus, Japan).

### Animals and lymphocyte isolation

Forty-five sexually mature 12-week-old male Swiss Webster (SW) mice weighing 25-30 g each were obtained from the Central Animal House of the Faculty of Pharmacy at King Saud University. All animal procedures were performed in accordance with the standards set forth in the Guidelines for the Care and Use of Experimental Animals by the Committee for the Purpose of Control and Supervision of Experiments on Animals (CPCSEA) and the National Institutes of Health (NIH). The study protocol was approved by the Animal Ethics Committee of the Zoology Department, College of Science, King Saud University. All animals were allowed to acclimatize in metal cages inside a well-ventilated room for 2 weeks prior to the experiment. The animals were maintained under standard laboratory conditions (a temperature of 23°C, a relative humidity of 60-70% and a 12-hour light/dark cycle) and were fed a diet of standard commercial pellets and given water *ad libitum*. The animals were divided into 3 experimental groups (n = 15/group): group I was a control group that was subcutaneously injected with PBS, group II was subcutaneously injected with WEV (50 ng/ml for 12 hrs) and group III was subcutaneously injected with WEV+NP (20 ng/ml for 12 hrs). Lymphocytes were isolated from the animals' blood using Ficoll-Paque density gradients. The remaining red blood cells were osmotically lysed using ACK buffer. The cells were washed with phosphate-buffered saline (PBS), counted using the Trypan blue exclusion test, and cultured in R-10 culture medium (complete RPMI 1640 medium supplemented with 10% FCS, 2 mM L-glutamine, 100 IU/ml penicillin, 100 μg/ml streptomycin, 1 mM sodium pyruvate, and 50 μM 2-mercaptoethanol).

### F-actin polymerization assay

The isolated blood lymphocytes were cultured for two hour in culture medium before the F-actin polymerization test. Intracellular F-actin polymerization was assessed as previously described [12]. Briefly, cells were harvested and resuspended (4 × 10^6^/ml) in HEPES-buffered RPMI 1640 at 37°C with or without CXCL12 (250 ng/ml). At the indicated times, cell suspensions (100 μl) were added to 400 μl of assay buffer containing 4 × 10^-7 ^M FITC-labeled phalloidin, 0.5 mg/ml L-α-lysophosphatidylcholine (both from Sigma-Aldrich) and 4.5% formaldehyde in PBS. The fixed cells were analyzed using flow cytometry, and the mean fluorescence intensity (MFI) was determined for each sample. The percentage change in MFI was calculated for each sample at each time point using the following formula: (1-(MFI before the addition of CXCL12/MFI after the addition of CXCL12) × 100.

### Immunoblotting

Whole-cell lysates were prepared from lymphocytes that were isolated from the control and WEV- and WEV+NP-treated mice in RIPA buffer (20 mM Tris-HCl, pH 7.5, 120 mM NaCl, 1.0% Triton X100, 0.1% SDS, 1% sodium deoxycholate, 10% glycerol, 1 mM EDTA and 1% protease inhibitor cocktail, Roche). Following centrifugation at 16,000 × *g *at 4°C for 15 min, the protein concentrations in the supernatants were determined using a protein assay kit (Bio-Rad, Hercules, CA). Equal amounts of whole-cell protein (50 μg) were mixed with reducing sample buffer (0.92 M Tris-HCl, pH 8.8, 1.5% SDS, 4% glycerol, and 280 mM 2-ME) and separated using discontinuous SDS-PAGE. Proteins were transferred onto nitrocellulose membranes using a Bio-Rad Trans-Blot electrophoretic transfer device, and the membranes were blocked for 1 h at room temperature with 1% BSA or 5% skim milk dissolved in TBS (20 mM Tris-HCl, pH 7.4, and 150 mM NaCl) supplemented with 0.1% Tween 20. The membranes were then incubated in the same blocking buffer with anti-phospho-ERK, anti-phospho-AKT, anti-phospho-IκBα, anti-phospho-p38MAPK or anti-β-actin antibodies (1:1,000; Cell Signaling Technology, Beverly, MA). The blots were thoroughly rinsed and then incubated with an HRP-labeled species-matched secondary antibody for another 1 h. Protein bands were detected using enhanced chemiluminescence reagents (ECL, SuperSignal West Pico Chemiluminescent Substrate, Perbio, Bezons, France), and the ECL signals were recorded on Hyperfilm ECL. To quantify band intensities, the films were scanned, saved as TIFF files and analyzed using NIH ImageJ software.

### CFSE proliferation assay

Isolated blood lymphocytes were harvested, washed twice in PBS and stained with 0.63 mM carboxyfluorescein diacetate succinimidyl ester (CFSE) (Molecular Probes, Eugene, OR) for 8 min at room temperature. Residual CFSE was removed by washing three times in PBS, and CFSE-labeled cells were seeded in 6-well plates, treated with or without a mitogen cocktail and grown for 4 days in cell culture medium. The CFSE fluorescence intensity was measured using FACS analysis.

### Statistical analysis

Data are expressed as the mean ± standard error of the mean (SEM). Significant differences among groups were analyzed using a one-way analysis of variance (for more than two groups) followed by Tukey's post-test using SPSS software, version 17. Differences were considered statistically significant at P < 0.05. *P < 0.05, WEV-treated vs control; #P < 0.05, WEV+NP-treated vs. control; +P < 0.05, WEV+NP-treated vs. WEV-treated groups.

## Results

### WEV affects the cell viability of breast cancer cells but not normal cells

**Figure 1:**
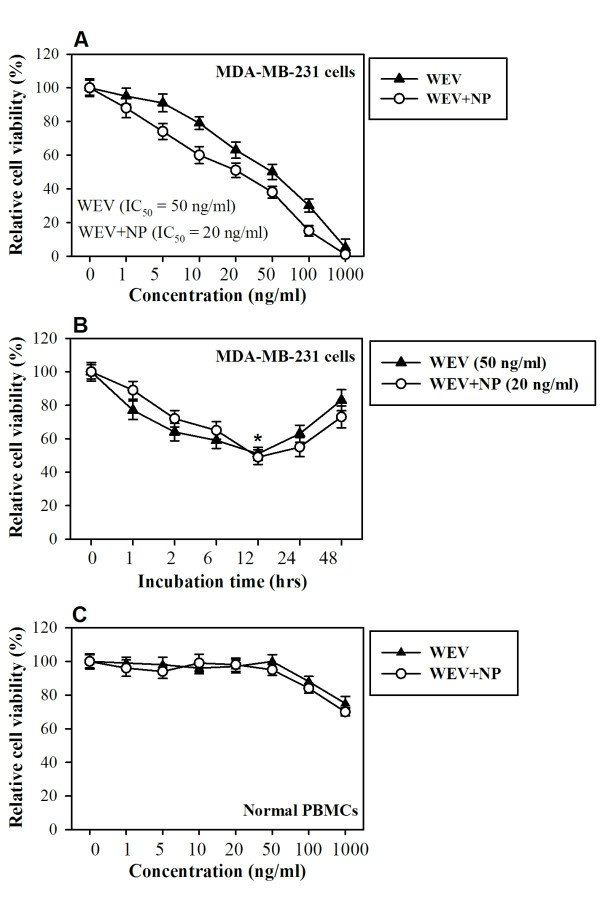
**Time- and dose-dependent responses of cell viability after WEV and WEV+NP treatment**. MDA-MB-231 cells were treated with WEV (closed triangles) or WEV+NP (open circles) at concentrations of 1, 5, 10, 20, 50, 100 and 1,000 ng/ml (**A**) for different incubation times (1, 2, 6, 12, 24 and 48 hr) (**B**). (**C**) Human PBMCs were treated with WEV or WEV+NP at concentrations of 1, 5, 10, 20, 50, 100 and 1,000 ng/mL for 12 hr. The experiments were performed in triplicate, and the results are expressed as the mean percentage of viable cells ± SEM.

The effect of WEV on the viability of MDA-MB-231 cells was assessed using the MTT uptake method. WEV inhibited the growth of MDA-MB-231 cells in a concentration- and time-dependent manner (Figure 1A &1B). The combination of WEV with NP (WEV+NP) enhanced the effect of WEV on the cancer cells. The maximal inhibitory effects of WEV and WEV+NP on cell viability were observed 12 hour after treatment with 50 ng/ml of WEV alone or with 20 ng/ml of WEV+NP. The effects of WEV and WEV+NP on the viability of normal human peripheral blood mononuclear cells (PBMCs) were also tested under the same conditions. Interestingly, the treatment of PBMCs with 50 ng/ml of WEV alone or 20 ng/ml of WEV+NP did not affect cell viability (Figure 1C). The survival of PBMCs after treatment with up to 1 μg/ml of WEV or WEV+NP was reduced by 25%. These experiments were performed in triplicate, and the results are expressed as the mean percentage of viable cells ± SEM. These results were confirmed using a Trypan blue exclusion test, which was used to determine cell viability and numerical cell counts simultaneously in all the experiments (data not shown).

### WEV combined with NP enhances CXCL12-mediated actin polymerization

**Figure 2:**
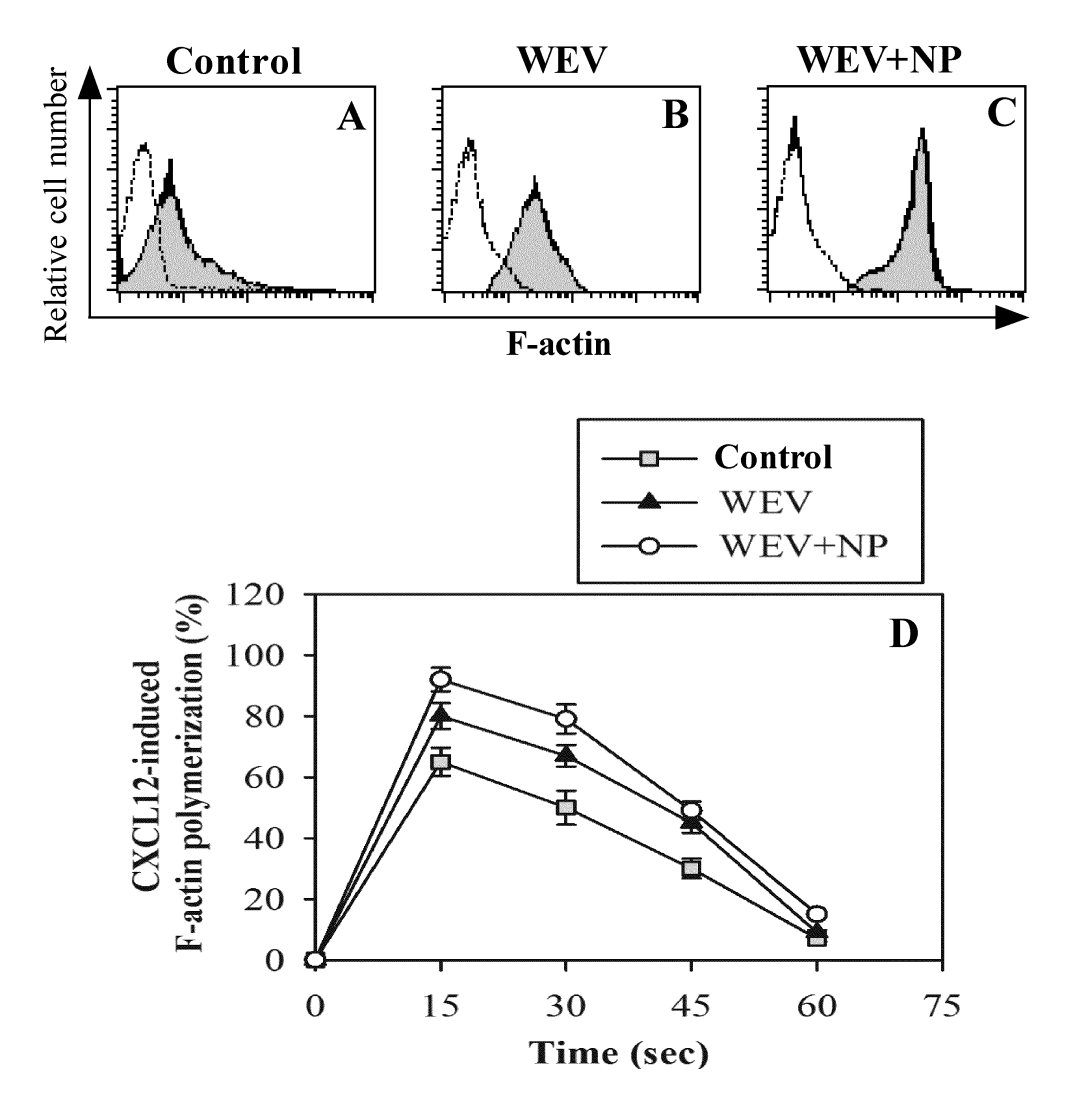
**WEV combined with NP enhanced CXCL12-mediated actin polymerization in mouse lymphocytes**. Isolated blood lymphocytes from the control (gray squares), WEV-treated (closed triangles) and WEV+NP-treated (open circles) mice were subjected to an F-actin polymerization assay after CXCL12 stimulation, and the results were quantified using flow cytometry. A representative experiment showing the degree of F-actin polymerization at 30 seconds is presented as gray histograms of cytometric data on lymphocytes from the control (**A**), WEV-treated (**B**) and WEV+NP-treated (**C**) mice. The results are expressed as the percentage change in MFI (n = 6) ± SEM (**D**), as described in the *Materials and Methods *section.

To analyze their effect on the viability of normal cells further, we investigated whether WEV alone or in combination with NP affected the normal functioning of lymphocytes *in vivo*. Three groups of mice (15 mice in each group) were injected with saline (the control group), WEV (50 ng/ml) or WEV+NP (20 ng/ml). Twelve hours post-injection, their blood lymphocytes were isolated, and the degree of CXCL12-mediated actin polymerization was determined. Actin and microtubules provide a dynamic cellular framework that orchestrates and ultimately controls cellular activation and cancer metastasis. Therefore, we monitored actin polymerization after CXCL12 stimulation in PBMCs isolated from the three groups of animals. The cells were stimulated every 15 sec with CXCL12 (250 ng/ml), and the degree of F-actin polymerization was determined using flow cytometry. The degree of F-actin polymerization at 30 seconds in one representative experiment is shown as the mean fluorescence intensity (MFI) as gray histograms representing the lymphocytes from the three groups of animals (Figure 2A, B &2C). The data from 10 individual mice in each group revealed that in the untreated control group, the percentage of F-actin polymerization in the PMBCs was 65 ± 4.6, 50 ± 5.6, 30 ± 3.2 and 7 ± 0.9 at 15, 30, 45 and 60 sec, respectively (gray squares) (Figure 2D). The percentage of F-actin polymerization was significantly increased in the PBMCs of the WEV-treated group (black triangles) to 80 ± 4.3, 67 ± 3.5, 45 ± 3.3 and 9 ± 0.8 at 15, 30, 45 and 60 sec, respectively. Most importantly, the percentage of F-actin polymerization was significantly increased in the PBMCs of the WEV-treated group (black triangles) to 92 ± 3.9, 79 ± 4.8, 49 ± 2.9 and 15 ± 0.7 at 15, 30, 45 and 60 sec, respectively, (P < 0.05; n = 10).

### WEV combined with NP increases CXCL12-mediated signaling through AKT, NF-κB and ERK but not p38MAPK

**Figure 3:**
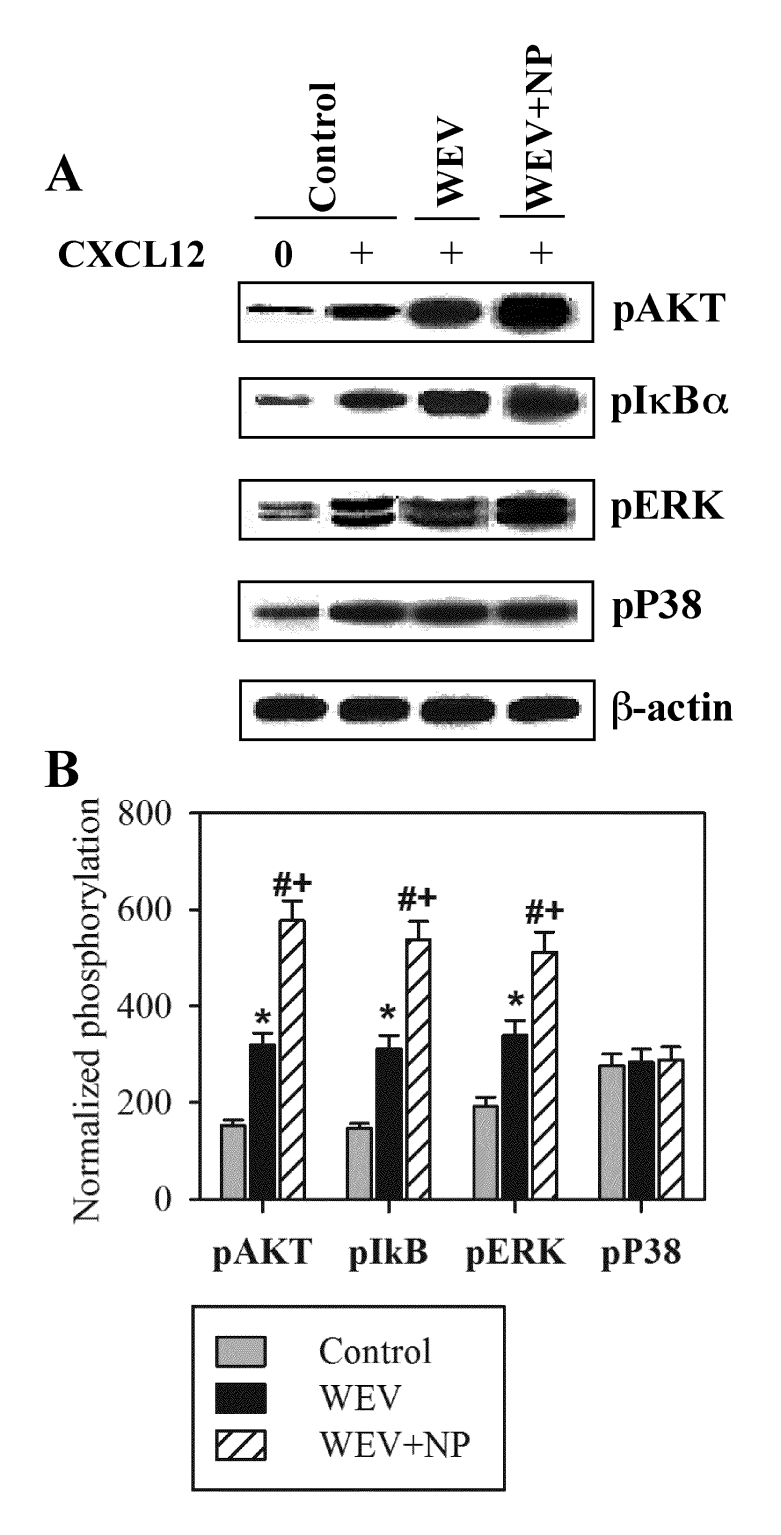
**Up-regulation of CXCL12-mediated PI3K/AKT, IκBα and ERK signaling in lymphocytes after treatment with WEV or WEV+NP**. The phosphorylation of AKT, IκBα, ERK and p38MAPK, which are the main signaling pathways downstream of CXCL12, was monitored in lymphocytes isolated from the three groups of mice using western blot analysis. (**A) **Protein bands from one representative experiment of the ten performed are shown for pAKT, pIκBα, pERK, p38MAPK and β-actin. **(B) **The phosphorylation of AKT, IκBα, ERK and p38MAPK was normalized to the total β-actin levels. The results are expressed as the mean ± SEM of normalized phosphorylation values in control (gray bars), WEV-treated (closed black bars) and WEV+NP-treated mice (hatched bars). *P < 0.05, WEV-treated vs. control; #P < 0.05, WEV+NP-treated vs. control; +P < 0.05, WEV+NP-treated vs. WEV-treated groups.

Lymphocytes are circulated continuously from the peripheral to the secondary lymphoid organs to recognize presented antigens (Ags), and this circulation is regulated by chemokines and their receptors. In the secondary lymphoid organs, chemokines and antigen recognition by lymphocytes mediate actin polymerization, cytoskeletal rearrangement, cell proliferation and differentiation to effector and Ag-specific memory cells. We therefore monitored the effect of the CXCL12 chemokine on the phosphorylation and activation of cytoplasmic transcription factors that regulate the proliferation of PBMCs in PBMCs isolated from the three groups of mice. Using antibodies against pAKT, pIκBα, pERK, p38MAPK and β-actin (as a control for the amount of protein loaded) in western blot analyses, we normalized the levels of phosphorylated AKT, IκBα, ERK and p38MAPK to the amount of total β-actin. In one representative experiment, we observed that stimulation with CXCL12 for 5 minutes enhanced the phosphorylation of AKT, IκBα, ERK and p38 MAPK. Moreover, the CXCL12-mediated phosphorylation of AKT, IκBα and ERK, but not P38MAPK, was clearly increased in PBMCs that were isolated from WEV- and WEV+NP-treated mice (Figure 3A). The results from 10 individual mice in each group showed that the normalized levels of phosphorylated AKT, IκBα and ERK induced by CXCL12 were significantly increased in the WEV-treated group compared with the control group (*P < 0.05). Moreover, the combination of WEV with NP significantly enhanced the effect of WEV on the CXCL12-mediated phosphorylation of AKT, IκBα and ERK compared with the control (^#^P < 0.05) and WEV-treated (^+^P < 0.05) groups (Figure 3B). In contrast, treatment with WEV or WEV+NP had no effect on the CXCL12-mediated phosphorylation of P38MAPK.

### WEV combined with NP enhances mitogen-induced cell proliferation

**Figure 4:**
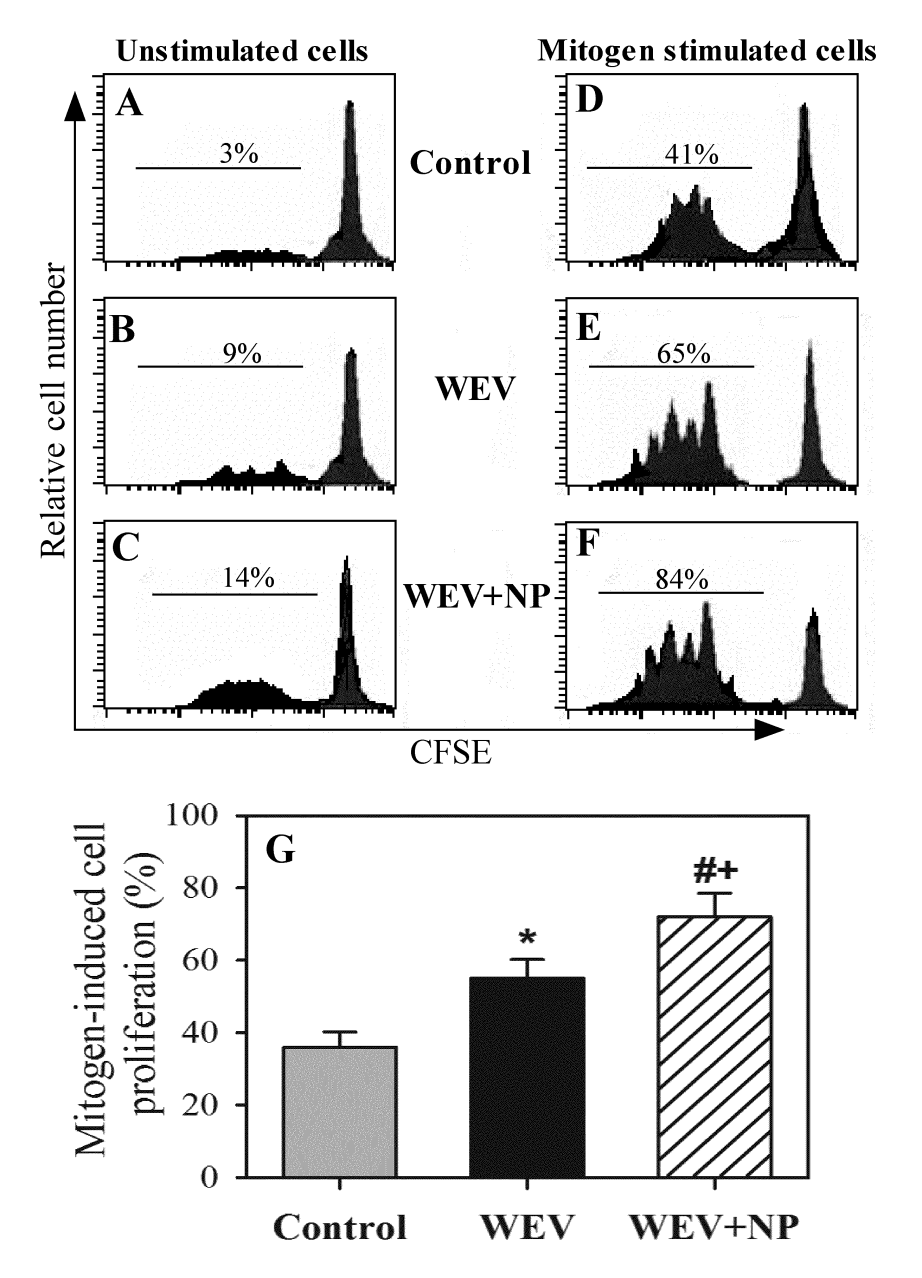
**WEV combined with NP enhanced lymphocyte mitogen activation and proliferation**. The ability of lymphocytes from the control, WEV-treated and WEV+NP-treated mice to proliferate spontaneously or in response to mitogen stimulation was evaluated using CFSE assays and flow cytometry. The isolated cells were labeled with CFSE, and the labeled cells were either left unstimulated (**A**, control; **B**, WEV; and **C**, WEV+NP) or stimulated with mitogen (**D**, control; **E**, WEV; and **F**, WEV+NP). The analysis of CFSE staining was performed after gating on viable cells. The percentage of proliferating cells (CFSE-lo) is indicated for each panel. One representative experiment of the ten is shown. (**G**) Data from 10 different experiments are expressed as the mean percentage of proliferating cells ± SEM for the control (gray bars), WEV-treated (closed black bars) and WEV+NP-treated (hatched bars) mice. *P < 0.05, WEV-treated vs. control; #P < 0.05, WEV+NP-treated vs. control; ^+^P < 0.05, WEV+NP-treated vs. WEV-treated groups.

Because treatment with WEV and WEV+NP enhanced CXCL12-mediated signaling, which regulates cell proliferation, we investigated whether the ability of PBMCs to proliferate in response to mitogen, a phenomenon important for the maintenance and survival of immune cells, was altered by treatment with WEV or WEV+NP. We stimulated PBMCs isolated from the three groups of mice with mitogen and determined the proliferative ability of lymphocytes after treatment with WEV or WEV+NP using CFSE proliferation and flow cytometry analyses. In one representative experiment, illustrated in Figure 4A, B &4C, we found that the percentage of spontaneously proliferating PBMCs was markedly increased, rising from 3% in the control group to 9% and 14% in the WEV- and WEV+NP-treated groups, respectively. When the cells were stimulated with mitogen, the percentage of proliferation was 41% in the control group versus 65% and 84% in WEV- and WEV+NP-treated groups, respectively (Figure 4D, E &4F). To generate the results shown in Figure 4G, we calculated the mitogen-induced specific proliferation of PBMCs by subtracting the spontaneous proliferation (i.e., the percentage of un stimulated cells in the absence of mitogen) from mitogen-induced proliferation (the percentage of mitogen-stimulated cells). Our results revealed that the proliferative capacity of PBMCs was significantly increased, changing from 36 ± 4.2% in the control group to 55 ± 5.2% and 72 ± 6.5% in the WEV- and WEV+NP-treated groups, respectively (*P < 0.05, WEV vs. control; ^#^P < 0.05, WEV+NP vs. control; ^+^P < 0.05, WEV+NP vs. WEV; n = 10).

## Discussion

Although many studies have investigated the anti-tumor and cytotoxic effects of snake venom on numerous types of cancer cells [13,14], little is known regarding its effects on breast cancer. Here, we investigated the effects of snake venom on the MDA-MB-231 cell line. Proliferation and survival are critically important for tumor growth and metastatic spreading; therefore, proliferation and survival constitute attractive targets for tumor therapy. First, we assessed the ability of WEV to arrest the growth of the MDA-MB-231 cell line, and we found that WEV affected the cell viability of breast cancer cells without inhibiting the viability of normal cells. The combination of WEV with NP (WEV+NP) enhanced the effect of WEV on the cancer cells. Our results agree with the results of Park et al. (2009) [14], who attributed this growth inhibition to apoptosis and cell cycle arrest. Interestingly, at the same concentrations, the venom neither alone nor in combination with nanoparticles induced the *in vitro *growth arrest of normal PBMCs. We next investigated how this venom might alter the biology of normal lymphocytes *in vivo*. Actin cytoskeletal reorganization is the primary mechanism of cell motility and is essential for lymphocyte migration to the secondary lymphoid organs, which is regulated by chemokines [12,15]. Therefore, we monitored actin polymerization in response to CXCL12 stimulation and found that WEV combined with NP enhanced CXCL12-mediated actin polymerization. Similarly, Oliva et al. (2007) [16] suggested that RGD-disintegrins isolated from snake venom were potent anti-metastatic agents that contributed to the inhibition of melanoma cell invasion through the involvement of the actin cytoskeleton. The control of microfilament actin remodeling thus represents a potential target for the development of anticancer drugs [17]. It has been reported that chemokines such as CCL20, CCL21 and CXCL12 induce actin polymerization and chemotaxis in B lymphocytes through the activation of PI3K/AKT, NF-κB, PLC, ERK and P38MAPK signaling [18,19]. Our data revealed that WEV combined with NP increased CXCL12-mediated signaling through AKT, NF-κB and ERK but not through p38MAPK. A previous study found that tungsten carbide-cobalt nanoparticles at 5 μg/cm^2 ^induced the production of reactive oxygen species (ROS) that activated AKT and ERK signal pathways in murine epidermal cells [20]. Additionally, in this study, WEV combined with NP enhanced mitogen-induced lymphocyte proliferation. Similar observations have been reported in the induction of endothelial cell proliferation, migration, and angiogenesis by the interaction of aggretin, a component of snake venom, with integrin α2β1, leading to the activation of PI3K and AKT [21]. Taken together, our data demonstrate a new effect of snake venom in combination with nanoparticles on the biological functioning of normal lymphocytes. However, the growth arrest of the breast cancer cell line by WEV and WEV+NP is more interesting. Therefore, in an ongoing work, we are studying the effects of WEV and WEV+NP on the growth arrest of cancer cell lines in an attempt to elucidate the molecular mechanism(s) by which this venom affects cancer cells.

## Abbreviations

CFSE: Carboxyfluorescein diacetate succinimidyl ester; CXCL12: CXC chemokine ligand 12; ERK: Extracellular signal-regulated kinase; IκBα: Inhibitor of nuclear factor kappa B alpha; NF-κB: Nuclear factor kappa B; PI3K: Phosphatidylinositol-3 kinase; PKB or AKT: Protein kinase B; WEV: *Walterinnesia aegyptia *venom; WEV+NP: *Walterinnesia aegyptia *venom combined with nanoparticles.

## Competing interests

All authors have read and agreed the contents of the manuscript and approved the submission. The authors declare no conflicts of interest, state that the manuscript has not been published or submitted elsewhere, state that the work complies with Ethical Policies of the Journal and the work has been conducted under internationally accepted ethical standards after relevant ethical review.

## Authors' contributions

GB put the study design, carried all the experiments, performed the statistical analysis, prepared the figures and drafted the manuscript, MKA was responsible for the extraction of snake venom and participated in the manuscript revision. AME was responsible for loading the venom on the silica nanoparticles. MD participated in the manuscript revision. All authors read and approved the final manuscript.
